# Genistein inhibits radiation-induced activation of NF-κB in prostate cancer cells promoting apoptosis and G_2_/M cell cycle arrest

**DOI:** 10.1186/1471-2407-6-107

**Published:** 2006-04-26

**Authors:** Julian J Raffoul, Yu Wang, Omer Kucuk, Jeffrey D Forman, Fazlul H Sarkar, Gilda G Hillman

**Affiliations:** 1 Department of Radiation Oncology, Barbara Ann Karmanos Cancer Institute, Wayne State University School of Medicine, Detroit, MI, 48201, USA; 2 Division of Hematology/Oncology, Department of Internal Medicine, Barbara Ann Karmanos Cancer Institute, Wayne State University School of Medicine, Detroit, MI, 48201, USA; 3 Department of Pathology, Barbara Ann Karmanos Cancer Institute, Wayne State University School of Medicine, Detroit, MI, 48201, USA; 4 Harper University Hospital, Detroit, MI, 48201, USA

## Abstract

**Background:**

New cancer therapeutic strategies must be investigated that enhance prostate cancer treatment while minimizing associated toxicities. We have previously shown that genistein, the major isoflavone found in soy, enhanced prostate cancer radiotherapy *in vitro *and *in vivo*. In this study, we investigated the cellular and molecular interaction between genistein and radiation using PC-3 human prostate cancer cells.

**Methods:**

Tumor cell survival and progression was determined by clonogenic analysis, flow cytometry, EMSA analysis of NF-κB, and western blot analysis of cyclin B1, p21^WAF1/Cip1^, and cleaved PARP protein.

**Results:**

Genistein combined with radiation caused greater inhibition in PC-3 colony formation compared to genistein or radiation alone. Treatment sequence of genistein followed by radiation and continuous exposure to genistein showed optimal effect. Cell cycle analysis demonstrated a significant dose- and time-dependent G_2_/M arrest induced by genistein and radiation that correlated with increased p21^WAF1/Cip1 ^and decreased cyclin B1 expression. NF-κB activity was significantly decreased by genistein, yet increased by radiation. Radiation-induced activation of NF-κB activity was strongly inhibited by genistein pre-treatment. A significant and striking increase in cleaved PARP protein was measured following combined genistein and radiation treatment, indicating increased apoptosis.

**Conclusion:**

A mechanism of increased cell death by genistein and radiation is proposed to occur via inhibition of NF-κB, leading to altered expression of regulatory cell cycle proteins such as cyclin B and/or p21^WAF1/Cip1^, thus promoting G_2_/M arrest and increased radiosensitivity. These findings support the important and novel strategy of combining genistein with radiation for the treatment of prostate cancer.

## Background

Prostate cancer (PCa) is an important public health concern in the United States. As our population ages, the number of patients with clinically significant PCa is expected to increase. In the United States, PCa is the most commonly diagnosed cancer in men as well as the second leading cause of male cancer deaths. The American Cancer Society estimates that in 2006 there will be 234,460 new cases of PCa and 27,350 men will die of the disease [1]. Localized PCa is sensitive to conventional radiotherapy using megavoltage photons (X-rays), yet residual disease often causes clinical relapse in a large proportion of patients [2,3]. While there is continuing debate on the impact of various treatment modalities on the survival of patients with different stages of PCa, the utilization of nutrition as an adjuvant therapy is an attractive idea. The use of dietary supplements, including soy, for cancer therapy has been recently reviewed [4,5]. To improve the local control and treatment of PCa, we have investigated the combination of genistein with conventional radiation treatment.

Genistein (4',5,7-trihydroxyisoflavone), the most abundant isoflavone found in soybeans, is believed to be a potent anticancer agent [6,7]. The interest in genistein stems from observations that increased soy consumption in Asian diets, resulting in increased serum isoflavone levels, has been associated with a decreased risk for PCa [8]. Genistein has an heterocyclic diphenolic structure similar to estrogen [9] and has demonstrated anti-tumor and anti-angiogenic activities [10,11]. Genistein was found to inhibit tyrosine protein kinases [12], topoisomerase I and II [13], and protein histidine kinase [14]. Genistein has also been shown to inhibit cell growth of tumor cell lines from various malignancies including breast, lung, melanoma, prostate, head and neck squamous cell carcinoma, leukemia and lymphoma [15-22].

We have previously shown that genistein inhibited the cell growth of androgen-dependent (LNCaP) and androgen-independent (PC-3) human prostate carcinoma cell lines [23]. Genistein affected the cell cycle and induced apoptosis, establishing it as a cytotoxic agent for PCa. We found that genistein induced G_2_/M cell cycle arrest leading to cell growth inhibition [23]. Cell growth inhibition was observed with concomitant down-regulation of cyclin B1, up-regulation of the p21^WAF1/Cip1 ^growth inhibitory protein, and induction of apoptosis [23]. We have also demonstrated that genistein augments radiation-induced cell killing of PC-3 prostate cancer cells *in vitro *[24]. Genistein combined with radiation significantly inhibited DNA synthesis, cell division, and cell growth compared to each modality alone [24].

We have also demonstrated *in vivo *that genistein potentiated inhibition of tumor growth by radiation in an orthotopic metastatic PC-3/nude mouse xenograft PCa tumor model [25]. Genistein combined with prostate tumor irradiation led to a greater control of the growth of the primary tumor and metastasis to lymph nodes than genistein or radiation alone, resulting in greater mouse survival [25]. These results suggest the potential for combining genistein with radiation for the treatment of localized PCa in humans.

The goal of our present study was to further elucidate the cellular and molecular interaction between genistein and radiation *in vitro*. We have investigated the effect of genistein and radiation on cell cycle progression and apoptosis and determined the optimal dose and time kinetics of each. We show that the potentiation of radiation-induced cell killing by genistein was optimal with the sequence of pre-treatment with genistein followed by radiation and continued exposure with genistein. This effect was also observed with other human tumor cell lines from various malignancies, indicating that our treatment strategy is not PCa cell-specific. The G_2_/M cell cycle arrest observed with genistein was enhanced by combination with radiation. This effect was associated with a greater up-regulation of the p21^WAF1/Cip1 ^growth inhibitory protein and down-regulation of cyclin B1 than that seen with genistein alone, resulting in a significant increase in apoptosis. Moreover, the inhibition of NF-κB DNA binding activity induced by genistein was also enhanced by combining genistein with radiation. We propose a mechanism of increased cell death in PCa cells pre-treated with genistein that may be dependent upon downregulation of radiation-induced NF-κB, thus driving cancer cells toward apoptotic versus survival pathways.

## Methods

### Tumor cell lines

The experiments were performed using the PC-3 human prostate carcinoma tumor cell line purchased from American Type Culture Collection (ATCC, Rockville, MD). PC-3 cells were cultured in F-12 K culture medium (CM) (Invitrogen, Carlsbad, CA) supplemented with 7% heat-inactivated fetal bovine serum, 2 mmol/L glutamine, 0.1 mmol/L non-essential amino acids, 10 mmol/L HEPES, and 100 U/mL penicillin/streptomycin. Human breast cancer cell line BR231 (MDA-MB-231) was purchased from ATCC. The human renal cell carcinoma (RCC) cell line KCI-18 was established in our laboratory from a primary renal tumor specimen obtained from a patient with papillary RCC (nuclear grade III/IV) [26]. The human RCC RC-2 cell line has been previously described [27]. These cell lines were cultured in Dulbecco's modified Eagle's medium (Invitrogen, Carlsbad, CA) with 4.5 g/L glucose supplemented with 10% fetal bovine serum, 2 mmol/L glutamine, 10 mmol/L HEPES, 100 U/mL penicillin/streptomycin and 50 μg/mL gentamicin. The cultures were incubated at 37°C in a humidified 5% CO_2 _incubator.

### Genistein treatment

Genistein was purchased from Toronto Research Chemicals (North York, Ontario, Canada) and dissolved in 0.1 mol/L Na_2_CO_3 _(Sigma, St. Louis, MO) to make a 10 mmol/L stock solution. Genistein was further diluted in CM to obtain concentrations of 15 μmol/L or 30 μmol/L. Control cells were incubated with equivalent dilutions of Na_2_CO_3 _in CM. Genistein treatment was administered when cells were 70% to 80% confluent.

### Radiation treatment

Cells in 15 mL tubes, T_25 _flasks, or T_75 _flasks were irradiated with photons using a ^60^Co unit (AECL Theratron 780). Tubes were placed at a depth of 2.6 cm in a specially machined lucite block of dimensions 10 cm × 20.3 cm × 12.8 cm [24]. The surface of the block was positioned at 46 cm from the source and tubes were irradiated with a horizontal 25 cm × 25 cm beam at a dose rate was -92 cGy min^-1 ^[24]. Flasks were irradiated from above with a vertical beam, 2.5 mm of polystyrene build up material was placed on top of the flasks and the surface of the build-up material was at a distance of 76 cm from the source. The dose rate was -32 cGy min^-1^.

### Analysis of cell survival by clonogenic assay

Cells were plated in T_25 _flasks at 0.5 × 10^6 ^cells/flask in CM. Three days later, 75% confluent cells were washed in CM and treated with genistein at a final concentration of 15 μmol/L in 5 mL CM. After 24 hr exposure to genistein, cells were removed using trypsin-EDTA (Invitrogen, Carlsbad, CA), counted and transferred to 15 mL conical tubes at 2 × 10^6 ^cells/5 mL CM for photon irradiation. Following irradiation, cells were plated in triplicate using 6-well plates. For comparison between each treatment group, the number of cells plated after genistein and/or radiation treatment was adjusted relative to untreated cells to predict a measurable survival fraction, as determined in pilot experiments. Based on these data, the number of cells plated in 2 mL CM were as follows: 500 cells/well for control, 1000 cells/well for genistein or radiation alone, and 3000 cells/well for genistein + radiation treatments. After plating, cells in respective treatment groups were supplemented with genistein at a final concentration of 15 μmol/L. Following 10–13 days incubation at 37°C in a 5% CO_2_/5% O_2_/90% N_2 _incubator, colonies were fixed and stained in 2% crystal violet in absolute ethanol, then counted. Clones of at least 50 cells were counted as one colony. The plating efficiency was calculated for each well by dividing the number of colonies by the original number of cells plated. The surviving fraction was normalized to control cell plating efficiency by dividing the plating efficiency of treated cells by that of control cells.

### Analysis of cell cycle progression

PC-3 cells were plated in T_25 _flasks at 1 × 10^6 ^cells/flask in CM. One day later, when 75% confluent, cells were washed in CM and treated with genistein at a final concentration of 15 μmol/L or 30 μmol/L in 5 mL CM. After 24 hr exposure to genistein, the cell monolayer in T_25 _flasks was irradiated with 3 Gy photons. On day 4 post-radiation, flasks were washed with Hanks' balanced salt solution (HBSS) and removed using trypsin-EDTA (Invitrogen, Carlsbad, CA). Cells were then washed in phosphate-buffered saline (PBS), counted and 0.5 × 10^6 ^cells/100 μl PBS buffer were fixed and permeabilized in 4.5 mL of 70% cold ethanol for 2 hr on ice. Cells were washed again in PBS then stained for 30 min at room temperature with 1 mL DNA fluorochrome solution containing 200 μg propidium iodide (Sigma, St. Louis, MO), 0.1% Triton X-100 (Sigma, St Louis, MO), and 2 mg DNase-free ribonuclease A (Sigma, St Louis, MO). Cells were analyzed using a FACScan flow cytometer (Becton Dickinson, Mountain View, CA).

### Analysis of protein expression by western blot

Cells were plated in T_75 _flasks at 1 × 10^6 ^cells/flask in CM. Seventy-five percent confluent cells were washed in CM then treated with genistein at a final concentration of 30 μmol/L in 10 mL CM. After 24 hr exposure to genistein, cells were irradiated with 3 Gy photons. After 1 hr, cells were removed using trypsin-EDTA (Invitrogen, Carlsbad, CA) and collected by centrifugation. Nuclear and cytoplasmic proteins were isolated using CelLytic™ NuCLEAR™ Extraction Kit (Sigma, St. Louis, MO) according to the manufacturer's protocol. Extracts were aliqouted, flash frozen in liquid nitrogen, and stored at -80°C for subsequent western blot analyses. Protein concentrations were determined according to Bradford using Protein Assay Kit I (Bio-Rad, Hercules, CA).

Western analysis was performed using 20 μg nuclear extracts as previously described [28]. Briefly, each sample was prepared with an equal volume of 2X loading dye (National Diagnostics, Atlanta, GA), subjected to 10% SDS-PAGE (.75 mm thick; 30% Acrylamide/Bis Solution 29:1) and transferred to a Hybond™ ECL™ nitrocellulose membrane (Amersham Pharmacia Biotech, Piscataway, NJ) using a semi-dry transfer apparatus (Bio-Rad, Hercules, CA). SDS-PAGE progression was monitored using dual color Precision Plus Protein™ Standards (Bio-Rad, Hercules, CA). Upon completion of SDS-PAGE, the region containing the protein(s) of interest was excised and prepared for western analysis while the remaining portion of the gel was stained with GelCode^® ^Blue Stain Reagent (Pierce, Rockford, IL) to ensure equal quantity of protein was loaded onto the gel. Western blot analysis was accomplished using manufacturer recommended dilutions of rabbit polyclonal anti-PARP (214/215) cleavage site specific antibody (BioSource, Camarillo, CA), mouse monoclonal anti-cyclin B1 antibody (D-11, Santa Cruz Biotechnology, Santa Cruz, CA), and mouse monoclonal anti-p21^WAF1/Cip1 ^antibody (187, Santa Cruz Biotechnology, Santa Cruz, CA). After incubation in recommended dilutions of goat anti-mouse IgG-HRP (Santa Cruz Biotechnology, Santa Cruz, CA) or goat anti-rabbit IgG-HRP (Cell Signaling Technology, Beverly, MA), membranes were incubated in SuperSignal^® ^West Pico Chemiluminescent Substrate (Pierce, Rockford, IL), exposed to CL-Xposure Film™ (Pierce, Rockford, IL) and developed using an All-Pro 100 Plus automated X-ray film processor (All-Pro Imaging Corporation, Hicksville, NY). Membranes were stripped using Restore™ buffer (Pierce, Rockford, IL) and reprobed with rabbit polyclonal anti-Rb antibody (C-15, Santa Cruz Biotechnology, Santa Cruz, CA) and developed as described above as an additional control for nuclear protein loading. The resultant bands were quantified using AlphaEaseFC™ imaging software (AlphaInnotech, San Leandro, CA).

### Analysis of NF-κB DNA binding activity

PC-3 cells were treated with 30 μmol/L genistein for 24 hr then irradiated with 3 Gy photons. After 30 min, cells were removed using trypsin-EDTA (Invitrogen, Carlsbad, CA) and collected by centrifugation. Nuclear proteins were isolated as previously described [29]. Briefly, the cell pellet was resuspended in 0.5 mL lysis buffer (10 mmol/L Tris-HCl, pH 7.5; 5 mmol/L MgCl_2_; 0.05% Triton X-100) and lysed with 20 strokes in a 1 mL Dounce homogenizer. The homogenate was centrifuged at 10,000 g for 15 minutes at 4°C. The pellet was resuspended in equal volume of nuclear extraction buffer A (10 mmol/L Tris-HCl, pH 7.4; 5 mmol/L MgCl_2_) and nuclear extraction buffer B (1 mol/L NaCl; 10 mmol/L Tris-HCl, pH 7.4; 4 mmol/L MgCl_2_). The resuspended pellet was incubated on ice for 30 min and then centrifuged at 10,000 g for 15 min at 4°C. The supernatant containing the nuclear proteins was removed and 80% glycerol was added to a final glycerol concentration of 20% (vol/vol). Protein concentrations were determined using the bicinchoninic acid (BCA) protein assay (Pierce, Rockford, IL).

NF-κB DNA binding activity was determined by electrophoretic mobility shift assay (EMSA) as previously described [29]. Briefly, 10 μg nuclear extract was incubated with ^32^P-labeled and purified NF-κB consensus double-stranded oligonucleotide and 0.25 mg/mL poly(dI- dC) in 5X binding buffer (20% glycerol, 5 mmol/L MgCl_2_, 2.5 mmol/L EDTA, 2.5 mmol/L DTT, 250 mmol/L NaCl, 50 mmol/L Tris-HCl, pH7.5). After incubation at room temperature for 30 min, samples were loaded on a pre-run 8% polyacrylamide gel and run at 30 mA for 45 min. The gel was dried, exposed to X-ray film overnight at -80°C, then developed using an All-Pro 100 Plus automated X-ray film processor (All-Pro Imaging Corporation, Hicksville, NY). The resultant bands were quantified using AlphaEaseFC imaging software (AlphaInnotech, San Leandro, CA). Anti-Rb immunoblotting with nuclear protein was performed as a loading control as described above.

### Statistical analysis

Comparisons of survival fractions in the clonogenic assays among the various treatment groups were analyzed by two-tailed unpaired Student's *t*-Test. Comparisons between means in the western blot and EMSA assays among the various treatment groups were analyzed by two-tailed Student's *t*-Test for independent samples. A *p-*value less than 0.05 was considered statistically significant.

## Results

### Enhanced cell growth inhibition of prostate carcinoma cells and other tumor cell lines by genistein and radiation

**Figure 1:**
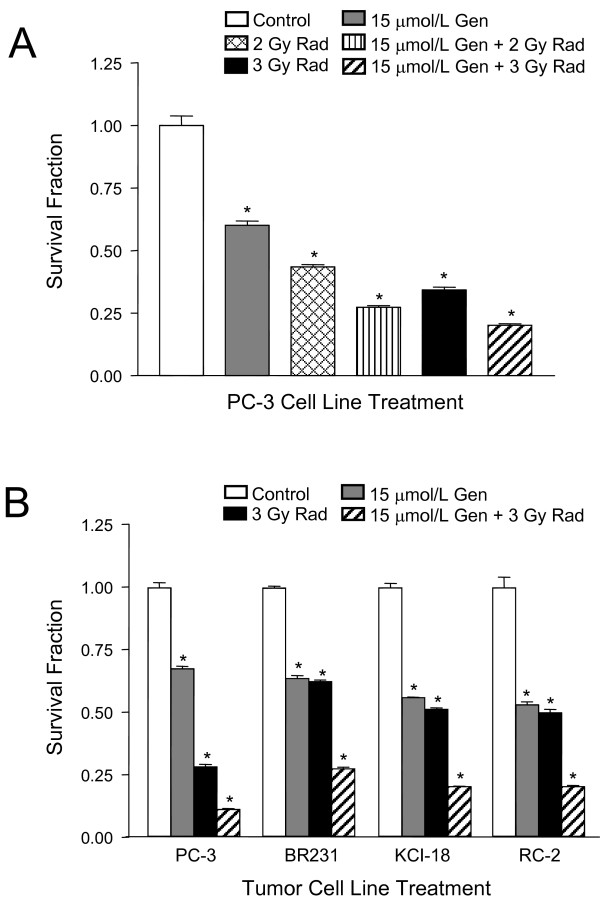
**Effect of genistein and radiation treatment on the survival fraction of human tumor cell lines**. **(A) **PC-3 prostate cancer cells were treated with 15 μmol/L genistein for 24 hr then irradiated with 2 or 3 Gy photons. After radiation, cells were plated in a clonogenic assay. **(B) **Cells from the PC-3 (prostate cancer), BR-231 (breast cancer), and KCI-18 and RC-2 (renal cell carcinoma) cell lines were treated with 15 μmol/L genistein for 24 hr then irradiated with 3 Gy photons and plated in a clonogenic assay. Colonies were stained with crystal violet and counted after 11–13 days incubation. The mean survival fraction ± S.E.M. of triplicate wells was normalized to control cells based on plating efficiency. (*): value statistically significant from control at *p *< 0.05. Combined treatment was significantly greater (*p *< 0.05) than genistein or radiation alone.

Doses of 15 μmol/L genistein and 3 Gy radiation were selected based on previous dose titration experiments [24]. Treatment of PC-3 cells with 15 μmol/L genistein or 3 Gy radiation alone promoted approximately 50% inhibition in cell growth, while optimal cell killing was observed after pre-treatment with 15 μmol/L genistein combined with low-dose photon radiation [24]. To confirm this observation, we have repeated the clonogenic assay with an adjusted number of cells plated after genistein and/or radiation treatment in order to predict a measurable survival fraction that permitted enhanced comparisons between single and combined treatments (see Methods section). PC-3 cells were treated with 15 μmol/L genistein for 24 hr then irradiated with 2 Gy or 3 Gy photon radiation. To evaluate the long-term effect of the single or combined treatments, cells were plated in a 10 day clonogenic assay in the presence of 15 μmol/L genistein. Genistein augmented cell growth inhibition induced by 2 Gy or 3 Gy radiation to 72% (*p *< 0.005) and 80% (*p *< 0.001), respectively compared to 55% and 65% with 2 Gy or 3 Gy radiation alone; whereas, genistein alone caused only 40% inhibition (Fig. 1A). Based on these data, the conditions of 15 μmol/L genistein and 3 Gy photon radiation were selected for continuation of the studies.

To assess whether the potentiation of radiation-induced cell killing by pre-treatment with genistein is not a phenomenon restricted to PC-3 cells, additional human tumor cell lines were tested (Fig. 1B). The response of PC-3 cells to genistein and radiation was compared to the human breast cancer cell line MDA-MB-231 (BR231) and also two renal cell carcinoma cell lines KCI-18 and RC-2. Cells were pre-treated with 15 μmol/L genistein for 24 hr, then irradiated with 3 Gy photon radiation and plated in a clonogenic assay in the presence of 15 μmol/L genistein. BR231, KCI-18 and RC-2 cell lines showed a comparable inhibition of cell growth when treated with genistein alone, but a lower response to radiation compared to PC-3 cells (Fig. 1B). However, the genistein combined with radiation caused a significant increase in inhibition of colony formation (73–80%, *p *< 0.05) compared to genistein (33–47%, *p *< 0.05) or radiation alone (40–50%, *p *< 0.05) in the three cell lines as observed for PC-3 cells (Fig. 1B).

### Sequence and exposure of genistein and radiation treatment

**Figure 2:**
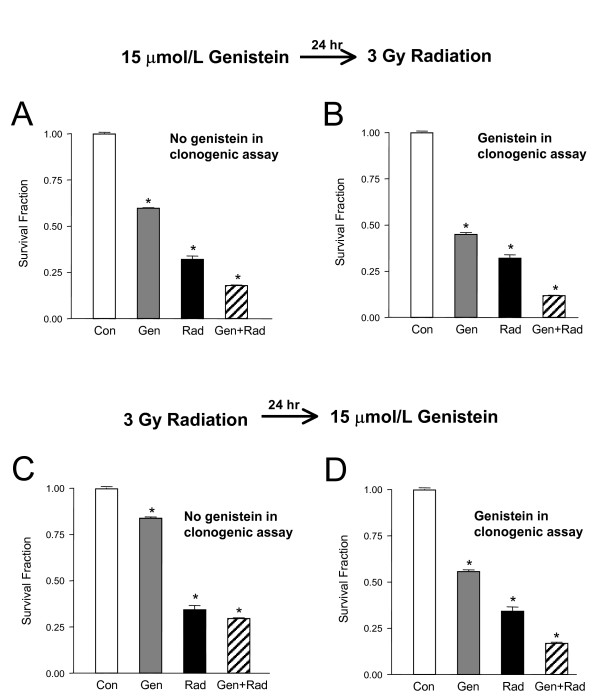
**Determination of the most effective combined treatment sequence in PC-3 cells**. PC-3 cells were analyzed by clonogenic assay to determine the most effective combined genistein + radiation treatment sequence. **(A) **PC-3 cells treated with 15 μmol/L genistein for 24 hr then irradiated with 3 Gy photons and plated in clonogenic assay. **(B) **PC-3 cells treated with 15 μmol/L genistein for 24 hr then irradiated with 3 Gy photons, followed by plating and continued treatment with 15 μmol/L genistein throughout clonogenic assay. **(C) **PC-3 cells irradiated with 3 Gy photons and 24 hr later treated with 15 μmol/L genistein for 24 hr then plated in clonogenic assay. **(D) **PC-3 cells irradiated with 3 Gy photons, and after 24 hr treated with 15 μmol/L genistein for 24 hr followed by plating and continued treatment with 15 μmol/L genistein throughout clonogenic assay. The colonies were stained and counted after 10 days incubation at 37°C in a 5% CO_2_/5% O_2_/90% N_2 _incubator. Data are presented as the mean survival fraction (± S.E.M.) of triplicate wells as normalized to control cells based on plating efficiency. (*): value statistically significant from control at *p *< 0.05.

To address the effect of the sequence and exposure of genistein relative to radiation, we have either pre-treated PC-3 cells with 15 μmol/L genistein for 24 hr followed by 3 Gy photon irradiation or pre-treated the cells with radiation and after 24 hr, treatment with 15 μmol/L genistein for 24 hr. Cells were then plated for the clonogenic assay in the presence or absence of 15 μmol/L genistein for 10 days. The sequence of genistein followed by radiation showed a greater cell growth inhibition with continuous exposure to genistein (88%, *p *< 0.05) than in the absence of genistein in the clonogenic assay (82%) (Fig. 2A,B). The effect of genistein alone was also more pronounced with continued exposure (55% inhibition vs. 40% without genistein, *p *< 0.05); whereas, the effect of radiation remained at the level of 68% inhibition (Fig. 2A,B). This sequence of genistein followed by radiation showed a greater effect on cell growth inhibition than the reverse sequence of radiation followed by genistein (*p *< 0.05) (Fig. 2C,D). Even in this reverse sequence, the effect of genistein alone or combined with radiation was greater with continued exposure of the cells to genistein compared to radiation alone and genistein + radiation (*p *< 0.05) (Fig. 2C,D).

### Analysis of cell cycle progression after treatment with genistein and radiation in PC-3 cells

**Figure 3:**
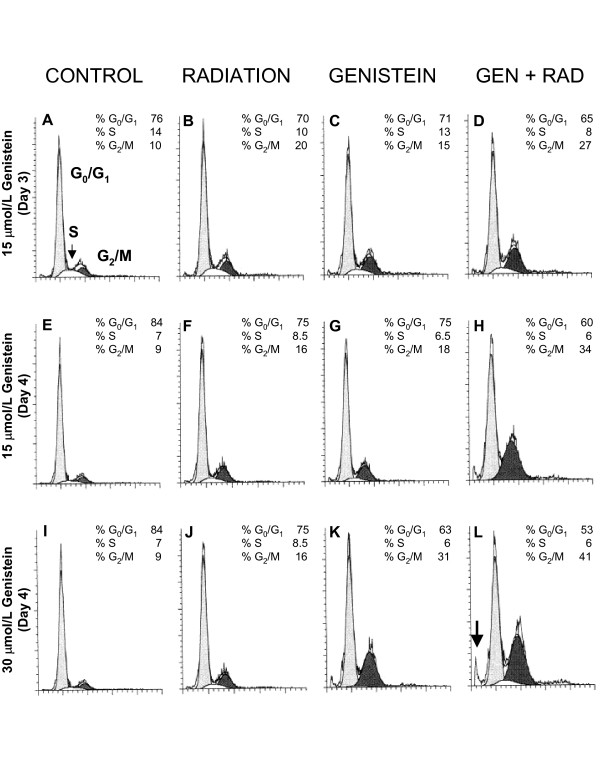
**Cell cycle analysis of PC-3 cells treated with genistein and radiation**. PC-3 cells were treated with either 15 μmol/L or 30 μmol/L genistein for 24 hr, 3 Gy photon radiation, or 15 μmol/L or 30 μmol/L genistein for 24 hr followed by 3 Gy photon radiation. On days 3 and 4 post-radiation, cells were processed for DNA content and cell cycle progression was analyzed by flow cytometry. (Arrow in Fig. 3L): indicates pre-G_0_/G_1 _apoptotic peak.

To investigate the molecular mechanism involved in potentiation of radiation-induced cell killing by genistein, we analyzed cell cycle progression and expression of molecules known to affect cell cycle regulation. PC-3 cells were treated for 24 hr with 15 μmol/L or 30 μmol/L of genistein then irradiated with 3 Gy photons. On Day 3 and 4 after radiation, separate flasks of cells were processed for DNA content and cell cycle analysis as described in the Methods section. From Day 3 after radiation, changes were observed in cell cycle analysis induced by genistein, radiation or both combined, showing an increased trend of cells in G_2_/M (Fig. 3A–D). Cell arrest in G_2_/M phase was more prominent by Day 4 after radiation (16%, Fig. 3J) and with a higher dose of 30 μmol/L genistein (31%, Fig. 3K) compared to 9% in control cells (Fig. 3I). Cells treated with genistein and radiation showed a further increase to 41% cells in G_2_/M cells (Fig. 3L) and a concomitant decrease in cells in G_0_/G_1 _phase from 84% to 53% (Fig. 3L). An increase in pre-G_0_/G_1 _peak of apoptotic cells was also noted (arrow, Fig. 3L). The percentage of cells in S phase remained at the level of 6–8%.

**Figure 4:**
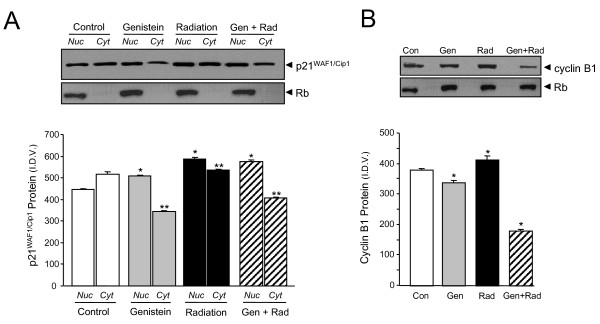
**Effect of genistein and radiation treatment on p21^WAF1/Cip1 ^and cyclin B1 expression in PC-3 cells**. PC-3 cells were treated with 30 μmol/L genistein for 24 hr then irradiated with 3 Gy photons. Nuclear and cytoplasmic proteins were extracted 1 hr post-radiation. The relative level of **(A) **p21^WAF1/Cip1 ^and **(B) **cyclin B1 protein in 20 μg of nuclear extract was determined by western blot analysis. Data are presented as the mean integrated density value (I.D.V.) of 3 separate experiments (± S.E.M.). (Con): control, untreated-cells; (Rad): cells irradiated with 3 Gy photons; (Gen): cells treated with 30 μmol/L genistein; (Gen + Rad): cells pre-treated with 30 μmol/L genistein for 24 hr then irradiated with 3 Gy photons. Retinoblastoma protein (Rb) was used as a nuclear protein loading control. (*): nuclear value statistically significant from control at *p *< 0.05; (**): cytoplasmic value statistically significant from control at *p *< 0.05.

Based on these data, we measured the expression of p21^WAF1/Cip1 ^and cyclin B1, two molecules involved in cell cycle progression. For these short-term molecular studies, and those described below, PC-3 cells were treated with 30 μmol/L genistein for 24 hr, followed by 3 Gy irradiation and processed for extraction of nuclear and cytoplasmic proteins at 1 hr post-irradiation. Following genistein and radiation, the expression of p21^WAF1/Cip1 ^showed a 1.5-fold increase in nuclear expression associated with 26% decrease in cytoplasmic expression (Fig. 4A). This pattern was comparable to that observed with genistein alone, although at a lower extent (1.1-fold increase in nuclear expression) (Fig. 4A). In contrast, radiation alone showed an significant increase in the expression of p21^WAF1/Cip1 ^protein both in the nuclear and cytoplasmic fractions (Fig. 4A). Genistein combined with radiation caused a 55% decrease in cyclin B1 expression in the cell nucleus compared to control cells (Fig. 4B). This decrease was greater than that observed with genistein alone (14% at 24 hr post-treatment) (Fig. 4B). No striking effect was observed with radiation alone when cells were tested at 1 hr post-radiation.

### Inhibition of radiation induced NF-κB activation by pre-treatment with genistein and induction of apoptosis in PC-3 cells

**Figure 5:**
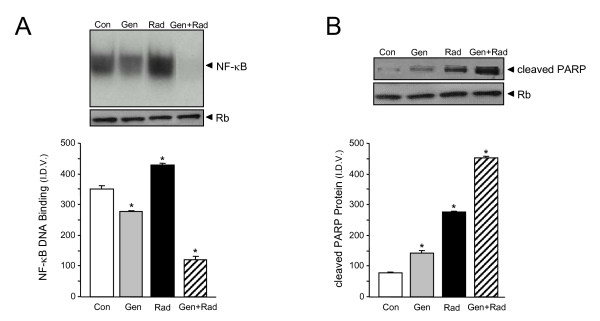
**Inhibition of radiation induced NF-κB activation and increased apoptosis by pre-treatment with genistein in PC-3 cells**. **(A) **PC-3 cells were treated with 30 μmol/L genistein for 24 hr then irradiated with 3 Gy photons. At 30 min post-radiation, cells were processed for isolation of nuclear protein. NF-κB DNA binding activity in 10 μg of nuclear extract was determined using EMSA. The level of DNA binding activity is expressed as the mean integrated density value (I.D.V.) from 3 separate experiments (± S.E.M.). **(B) **The level of the 85-kDa cleaved PARP protein in 20 μg of nuclear extract was determined by western blot analysis. Data are presented as the mean integrated density value (I.D.V.) of 3 separate experiments (± S.E.M.). (Con): control, untreated-cells; (Rad): cells irradiated with 3 Gy photons; (Gen): cells treated with 30 μmol/L genistein; (Gen + Rad): cells pre-treated with 30 μmol/L genistein for 24 hr then irradiated with 3 Gy photons. Retinoblastoma protein (Rb) was used as a nuclear protein loading control; (*): value statistically significant from control at *p *< 0.05.

We have previously demonstrated that apoptosis induced by genistein alone in PC-3 cells was associated with a decrease in NF-κB DNA binding activity [29], a well-known transcription factor critically involved in the survival of cells. Preliminary studies showed that radiation at 3 Gy or 5 Gy of PC-3 cells caused increased NF-κB DNA binding activity detectable by 30 min post-radiation, persisted up to 3 hr post-radiation, and then a decrease was observed. Based on these data suggesting that activation of NF-κB was an early event in response to radiation, the conditions for testing NF-κB activity in cells treated with genistein and radiation were selected. Cells were treated with 30 μmol/L genistein for 24 hr, followed by 3 Gy irradiation and harvested at 30 min post-radiation and nuclear proteins were extracted for EMSA analysis of NF-κB DNA binding activity. An significant increase (23%, *p *< 0.0009) in NF-κB was observed at 30 min post-irradiation whereas genistein-treated cells exhibited a significant decrease (21%, *p *< 0.0015) in NF-κB DNA binding activity (Fig. 5A). Pre-treatment of PC-3 cells with genistein for 24 hr followed by radiation showed that NF-κB DNA binding activity was significantly inhibited (66% decrease, *p *< 0.00004) (Fig. 5A). This NF-κB DNA binding inhibition induced by genistein combined with radiation persisted for 1 hr and 3 hr post-radiation (data not shown), suggesting its mechanistic role for enhanced apoptosis. Furthermore, the inhibition of NF-κB DNA binding activity by genistein and radiation was consistent with western blot analysis, with these cells showing a 21% increase in NF-κB p65 cytoplasmic protein expression compared to control, untreated cells (data not shown).

To test whether the increased NF-κB DNA binding inhibition observed in PC-3 cells treated with genistein and radiation resulted in increased apoptosis, we analyzed the expression of the 85-kDa cleaved PARP protein, a marker for detecting apoptotic cells. Cells were treated with 30 μmol/L genistein for 24 hr, followed by 3 Gy irradiation and processed for extraction of nuclear proteins at 1 hr post-irradiation. Cleaved PARP expression was significantly and strikingly enhanced by genistein combined with radiation as demonstrated by a 5.6-fold (*p *< 0.0002) increase in expression compared to 3.6-fold (*p *< 0.0002) with radiation alone and 1.8-fold (*p *< 0.004) with genistein alone, relative to control (Fig. 5B).

## Discussion

The use of soy isoflavones to potentiate conventional cancer treatment is a promising area for investigation. Our laboratory has previously shown that pre-treatment with genistein, the major isoflavone in soy, enhanced radiation-induced cell killing of PC-3 human PCa cells *in vitro *[24]. In the current study, we investigated the mechanism of interaction between genistein and radiation *in vitro *at the cellular and molecular levels. Using a highly calibrated clonogenic assay, we confirmed our previous findings [24] showing that genistein combined with radiation caused 80% inhibition in the cell survival fraction compared to 40% with genistein at 15 μmol/L and 65% with 3 Gy photon radiation. Furthermore, we have demonstrated that the combination of genistein and radiation caused greater cell killing in both human breast and renal cancer cell lines than each modality alone, suggesting that this effect was not restricted to PC-3 cells and that this combined modality may also be applied towards the treatment of other cancers. To get an optimal effect, continuous exposure of the cells to genistein before and after radiation was needed. Our data indicate that the sequence of genistein followed by radiation and continuous exposure of genistein result in the most effective conditions for the combined cancer treatment.

We have previously shown that genistein treatment of PC-3 cells resulted in G_2_/M cell cycle arrest and altered the expression of two cell cycle regulatory proteins, the cyclin-dependent kinase (CDK) inhibitor p21^WAF1/Cip1 ^and cyclin B1 [23]. Cell cycle analysis confirmed that either genistein or radiation alone promote a decrease in the percentage of cells in G_0_/G_1 _and a concomitant increase in the percentage of cells in G_2_/M. A more significant G_2_/M cell cycle arrest was induced by pre-treatment with genistein followed by radiation compared to each modality alone, an effect which was both dose- and time-dependent. Cells respond to DNA damaging agents by activating cell-cycle checkpoints. Both genistein and radiation were independently found to cause late G_2_/M cell accumulation measurable 4 days after treatment in the current study and in previous studies [23,30]. Cells in the G_2_/M phase of the cell cycle have been shown to be more radiosensitive than cells in other phases of the cell cycle [30,31]. Pre- treatment with genistein does arrest cells in G_2_/M phase and thus could increase their radiosensitivity resulting in increased cell killing in addition to the direct cytotoxic effects of genistein and radiation. This interaction is in direct agreement with our observation that increased killing is optimal with the sequence of genistein pre-treatment followed by radiation compared to the reverse sequence.

The cell cycle regulatory molecule p21^WAF1/Cip1 ^is a member of Cip/Kip family of cyclin-dependent kinase inhibitors (CKIs) involved in cell cycle and apoptosis regulation [32-34]. Under cellular stress, p21^WAF1/Cip1 ^expression is increased through p53-dependent and -independent pathways [34]. As shown in our studies, genistein or radiation alone induced upregulation of p21^WAF1/Cip1 ^protein in PC-3 cells, although they are p53 defective [35]. Moreover, higher nuclear expression concomitant with decreased cytoplasmic p21^WAF1/Cip1 ^expression were found in genistein pre-treated PC-3 cells exposed to radiation, suggesting nuclear translocation. Previous studies have shown that increased levels of p21^WAF1/Cip1 ^in the nucleus led to inhibition of CDKs through its binding to the cyclin/CDK complexes including cyclin B/CDK complex [32-34]. The cyclin B1/CDK1 (also known as cdc2) complex is essential for progression of the cells through mitosis, therefore a decrease in cyclin B proteins can result in G_2_/M arrest [36]. Our data showed a greater decrease in nuclear cyclin B1 in PC-3 cells treated with genistein combined with radiation, whereas radiation did not affect cyclin B1 (when measured at 1 hr post-radiation).

Our findings on G_2_/M arrest in response to radiation combined with genistein in PC-3 cells corroborate previous studies in DU145 human prostate cancer cells [37] and in cervical cancer cells [38]. Our studies further address the role of the transcription factor NF-κB in the mechanism by which genistein enhances radiation-induced cell killing. Recent studies have shown that NF-κB, a major signaling molecule involved in the regulation of cellular proliferation and apoptosis [39-41], is constitutively activated in PCa and correlates with disease progression [42,43]. NF-κB promotes malignant behavior by suppressing apoptosis and stimulating transcription of proteins involved in cell cycle progression. NF-κB activation and nuclear translocation can lead to the synthesis of molecules critical for cell survival in response to stress. We and others have demonstrated that genistein inhibits the activation of NF-κB in multiple cancer cell lines [15], including PC-3 and LNCaP prostate cancer cells [29]. Furthermore, genistein pre-treatment abrogated the activation of NF-κB by the chemotherapeutic agents docetaxel or cisplatin [44]. Such an effect is demonstrated in our current study showing that genistein pre-treatment also completely inhibited radiation-induced activation of NF-κB. We also observed an increase in NF-κB p65 protein in the cytoplasm of cells treated with genistein and radiation, suggesting that NF-κB may not be translocated into the nucleus as we have shown in previous studies with genistein alone [29]. Recent studies have established a correlation between radioresistance of breast cancer cells and induction of both NF-κB and cyclin B1 and demonstrated that fractionated radiation induced cyclin B1 expression via an NF-κB-dependent mechanism [45]. The cyclin B1 decrease that we observed following genistein combined with radiation could be related to the inhibition of NF-κB DNA binding activity.

Recent studies have also demonstrated that p21^WAF1/Cip1 ^was induced in S/G_2_/M phases and correlated with NF-κB activation [46]. Our findings on the effect of radiation alone causing upregulation of p21^WAF1/Cip1^, G_2_/M arrest and NF-κB activation could follow the same pathway. However, the effect of genistein combined with radiation on induction and nuclear translocation of p21^WAF1/Cip1^, in addition to the observed increase in cells arrested in G_2_/M phase, may not occur via an NF-κB-dependent mechanism, as NF-κB DNA binding activity was in fact inhibited by the combined treatment. The association between NF-κB inhibition and the upregulation and nuclear translocation of p21^WAF1/Cip1 ^induced by genistein combined with radiation remains to be elucidated.

We propose a mechanism of increased cell killing by combined genistein and radiation treatment that is triggered by inhibition of NF-κB leading to altered transcription of regulatory cell cycle proteins such as cyclin B and/or p21^WAF1/Cip1^, thus promoting apoptotic cell death. Increased apoptotic cell death was confirmed by the observation of significantly elevated expression levels of cleaved PARP protein in cells treated with genistein and radiation, compared to each modality alone, demonstrating increased apoptotic cell death.

## Conclusion

Our current findings are consistent with the hypothesis that genistein pre-treatment sensitizes cancer cells to radiation-induced growth inhibition and apoptosis. We have also obtained similar results in recent studies demonstrating that genistein pre-treatment potentiates chemotherapy-induced tumor cell death both *in vitro *and *in vivo *[47-50]. Taken together, our studies demonstrate an important and novel strategy of combining conventional cancer treatment with nutrition and support the use of soy isoflavones in combination with radiation for the treatment of patients with prostate cancer.

## Competing interests

The author(s) declare that they have no competing interests.

## Authors' contributions

G.G.H. designed and supervised the study and prepared the manuscript. J.J.R. performed cell culture and molecular experiments including Western Blot and EMSA, and assisted with experimental design, data analysis, and preparation of the manuscript. Y.W. performed cell culture, clonogenic assays and flow cytometry experiments. F.H.S. participated in the design of the study. O.K. and J.D.F. are clinicians actively involved in this project and its translation to clinical trials. All authors read and approved the manuscript.

## Pre-publication history

The pre-publication history for this paper can be accessed here:
